# Chitin promotes antigen-specific Th2 cell-mediated murine asthma through induction of IL-33-mediated IL-1β production by DCs

**DOI:** 10.1038/s41598-018-30259-2

**Published:** 2018-08-06

**Authors:** Ken Arae, Hideaki Morita, Hirotoshi Unno, Kenichiro Motomura, Sumika Toyama, Naoko Okada, Tatsukuni Ohno, Masato Tamari, Keisuke Orimo, Yuko Mishima, Hajime Suto, Ko Okumura, Katsuko Sudo, Hiroshi Miyazawa, Haruhiko Taguchi, Hirohisa Saito, Kenji Matsumoto, Susumu Nakae

**Affiliations:** 1 Department of Immunology, Faculty of Health Sciences, Kyorin University, Tokyo, 181-8612 Japan; 2 Department of Allergy and Clinical Immunology, National Research Institute for Child Health and Development, Tokyo, 157-8535 Japan; 3 Department of Molecular Immunology, Graduate School of Medical and Dental Sciences, Tokyo Medical and Dental University, Tokyo, 113-8510 Japan; 4 Atopy Research Center, Juntendo University, Tokyo, 113-0033 Japan; 5 Animal Research Center, Tokyo Medical University, Tokyo, 160-8402 Japan; 6 Department of Medical technology, Faculty of Health Sciences, Kyorin University, Tokyo, 181-8612 Japan; 7 Laboratory of Systems Biology, Center for Experimental Medicine and Systems Biology, The Institute of Medical Science, The University of Tokyo, Tokyo, 108-8639 Japan; 8 Precursory Research for Embryonic Science and Technology (PREST), Japan Science and Technology Agency, Saitama, Japan

## Abstract

Chitin, which is a major component of house dust mites (HDM), fungi, crustaceans, etc., can activate immune cells, suggesting that it contributes to development of allergic disorders such as asthma. Although the pathophysiological sensitization route of asthmatic patients to allergens is considered via the respiratory tract, the roles of intranasally-administered chitin in development of asthma remain unclear. After ovalbumin (OVA) challenge, development of airway inflammation was profoundly exacerbated in mice sensitized with OVA in the presence of chitin. The exacerbation was dependent on IL-33, but not IL-25, thymic stromal lymphopoietin or IL-17A. Chitin enhanced IL-33-dependent IL-1β production by dendritic cells (DCs). Furthermore, chitin- and IL-33-stimulated DC-derived IL-1β promoted OVA-specific Th2 cell activation, resulting in aggravation of OVA-induced airway inflammation. These findings indicate the adjuvant activity of chitin via a new mechanism and provide important clues for development of therapeutics for allergic disorders caused by HDM, fungi and crustaceans.

## Introduction

Chitin, β-(1-4)-poly-N-acetyl-D-glucosamine, is widely distributed in nature as the second most abundant polysaccharide after cellulose. It is a common structural component in lower organisms: in the cell wall of pathogens such as bacteria and fungi, the sheath of parasitic nematodes, and the exoskeleton of crustaceans (crabs and shrimp) and insects^1–5^. Chitin is also present in the exoskeleton as well as feces of house dust mites (HDM), which is the most frequent and pervasive aeroallergen causing allergic diseases such as asthma and atopic dermatitis^4,6^.

Chitin and its derivatives have generally been considered harmless and non-allergic to humans, and some prostheses, including artificial skin and contact lenses, are made from chitin derivatives. However, it was shown that chitin can directly activate various types of immune cells, suggesting a role in induction of inflammatory diseases^4^. It was suggested that 1- to 10-μm diameter small chitin particles, which is a phagocytizable size for macrophages, can stimulate macrophages to produce IL-12 and TNF, followed by induction of IFN-γ production by lymphocytes such as NK cells, in mice^7,8^. In addition to TNF, appropriate-size chitin particles also induce IL-17 and IL-10 production by mouse macrophages via TLR2 and/or Dectin-1, which are considered to be receptors for chitin^9,10^. Moreover, intranasal or intraperitoneal administration of chitin beads to mice resulted in production of leukotriene B4 by macrophages and type 2 cytokines by ILC2, leading to development of airway eosinophilia even in the absence of acquired immune cells such as T cells and B cells, mast cells and STAT6-mediated signal transduction^11–13^.

In addition, chitin particles can enhance Th2 cell-mediated airway eosinophilia as an adjuvant, like alum does. That is, intraperitoneal administration of ovalbumin (OVA) to mice in the presence of chitin particles enhanced activation of ovalbumin (OVA)-specific Th2 cells as well as Th1 cells and Th17 cells, contributing to development of OVA-induced Th2 cell-mediated airway inflammation^14^. TLR2 and IL-17 are required for the effect of chitin as an adjuvant in the setting. However, it remains unclear how IL-17, which is produced by macrophages through interaction of chitin with TLR2, contributes to Th2 cell-mediated airway eosinophilia.

In general, the route of sensitization of patients with asthma to allergens such as HDM is considered to be via the respiratory tract and/or skin, but not the peritoneum. Therefore, the effect of intranasally–administered chitin on Th2 cell-mediated airway inflammation needs to be elucidated.

In the present study, we show that even via the intranasal route chitin acts as an adjuvant on OVA-induced Th2 cell-mediated airway inflammation, independently of IL-17. We also found a novel pathway for enhancement of Th2 cell-mediated airway inflammation by chitin: chitin induced IL-33 in the lungs, followed by augmentation of IL-33-mediated IL-1β production by dendritic cells (DCs). Subsequently, that IL-1β promoted OVA-specific Th2 cell expansion, resulting in aggravation of OVA-induced Th2 cell-mediated airway inflammation.

## Results

### Chitin shows potent adjuvant effect on allergic airway inflammation

**Figure 1:**
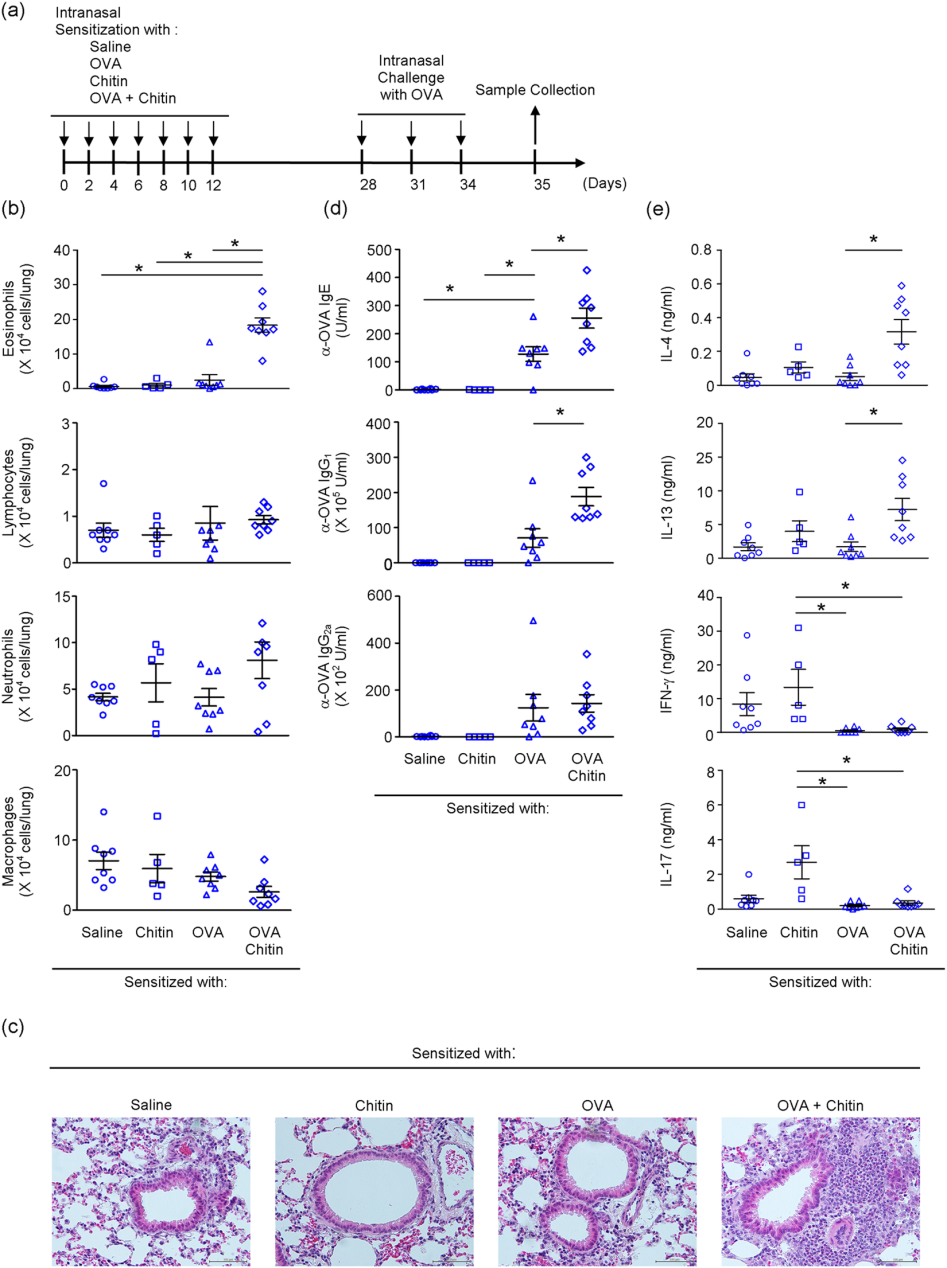
Chitin amplifies the development of OVA-induced airway inflammation. (**a**) Scheme of induction of allergic airway inflammation by OVA in mice sensitized intranasally with or without OVA ± chitin. BALB/c-wild-type mice were repeatedly intranasally sensitized with OVA, chitin, OVA + chitin or saline alone, and then challenged intranasally with OVA. Twenty-four h after the last OVA challenge, samples were collected for the following experiments. (**b**) The number of cells in BALFs. (**c**) Lung sections (hematoxylin and eosin staining, x 200). (**d**) Serum levels of OVA-specific IgE, IgG_1_ and IgG_2a_. (**e**) Cytokine levels in culture supernatants of spleen cells after *in vitro* restimulation with OVA. Data are pooled from 2 independent experiments and shown as the mean ± SE (b, d and e). 1-way ANOVA, *P < 0.05.

To investigate the effect of chitin on pulmonary immune responses, mice were treated intranasally with OVA or saline in the presence and absence of chitin (Fig. 1a). The mice were then intranasally challenged with OVA (Fig. 1a). One day after the last OVA challenge, the number of eosinophils, but not other types of cells, was profoundly increased in BALFs from mice sensitized with OVA in the presence of chitin compared with the mice sensitized with OVA alone, chitin alone or saline (Fig. 1b). Consistent with this, after the last OVA challenge, histological analysis showed airway inflammation in the mice sensitized with OVA in the presence of chitin, but not in mice sensitized with OVA alone, chitin alone or saline (Fig. 1c). In association with this, after the last OVA challenge, the levels of OVA-specific IgE, IgG_1_ and IgG_2a_ were significantly increased in sera from mice sensitized with OVA in comparison with mice treated with chitin alone or saline (Fig. 1d). The levels of OVA-specific IgE and IgG_1_, but not IgG_2a_, were approximately 2- to 3-fold increased in sera from mice sensitized with OVA in the presence of chitin compared with mice sensitized with OVA alone (Fig. 1d).

**Figure 2:**
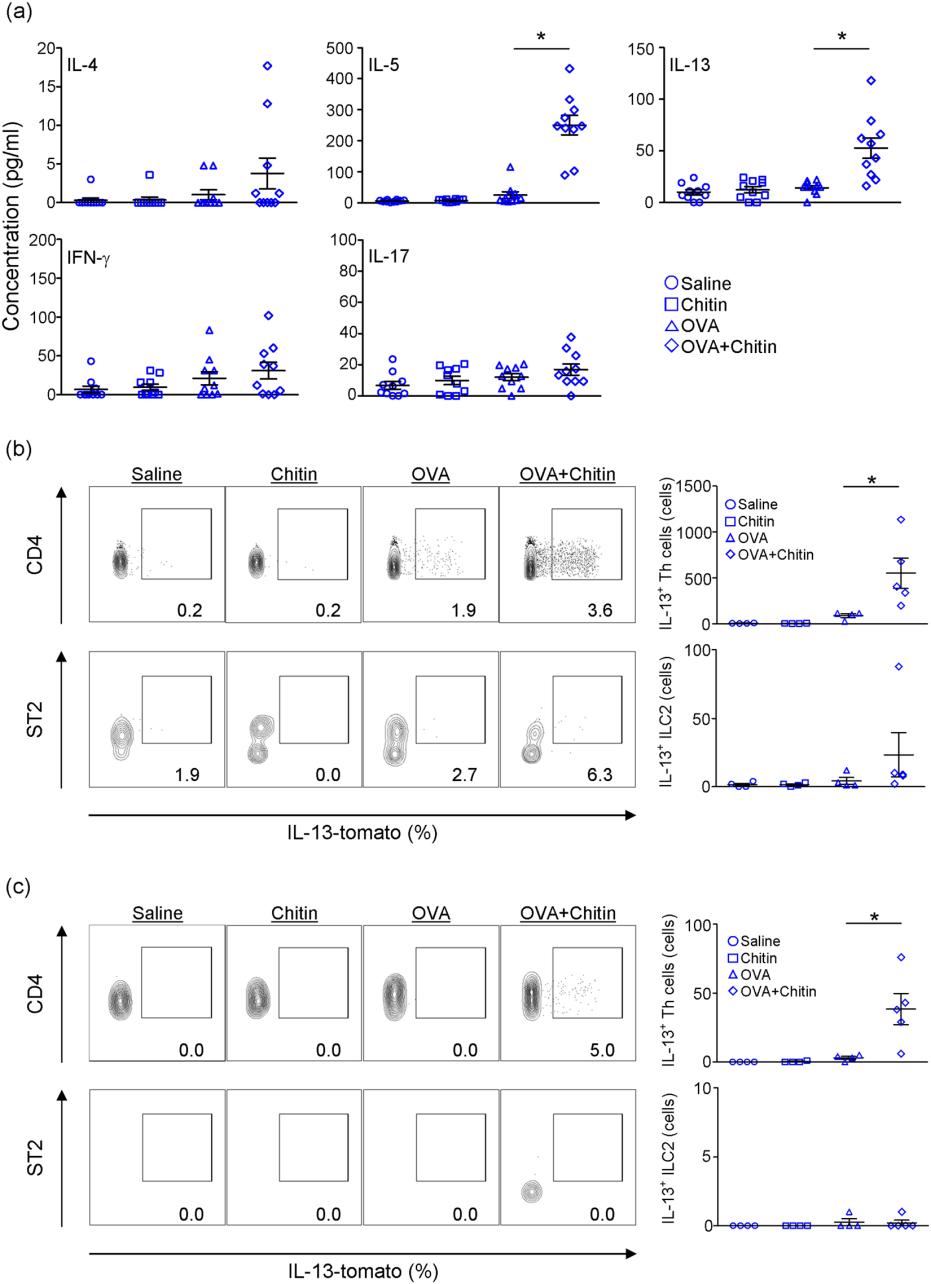
Chitin amplifies type 2 cytokine production. C57BL/6-wild-type (**a**) or C57BL/6-*Il13*^tomato/+^ (**b**,**c**) mice were repeatedly intranasally sensitized with OVA, chitin, OVA + chitin or saline alone, and then challenged intranasally with OVA. Twenty-four h after the last OVA challenge, samples were collected for the following experiments. (**a**) The levels of cytokines in BALFs were determined by ELISA. (**b**,**c**) The proportion and number of IL-13-tomato-positive cells in CD45^+^ CD4^+^ cells and CD45^+^ lin^-^ ST2^+^ cells in the lungs (**b**) and BALFs (**c**) were determined by FACS. Data are shown as the mean ± SE. 1-way ANOVA, *P < 0.05.

To investigate the effect of chitin on antigen-specific Th2 cell function during OVA-induced airway inflammation, spleen cells from mice sensitized with OVA in the presence and absence of chitin and from mice treated with chitin alone or saline were cultured in the presence of OVA. The levels of IL-4 and IL-13 in the culture supernatants of spleen cells from the OVA-sensitized mice were comparable to those from mice treated with chitin alone or saline (Fig. 1e). On the other hand, the supernatant IL-4 and IL-13 levels of mice sensitized with OVA in the presence of chitin were markedly increased compared with mice sensitized with OVA alone, chitin alone or saline (Fig. 1e). In contrast to the responses of the above Type-2-cytokines, the level of a Type-1-cytokine (IFN-γ) was significantly reduced in the culture supernatants of spleen cells from mice sensitized with OVA compared with mice treated with chitin alone or saline (Fig. 1e). However, this reduction was not influenced by the presence of chitin (Fig. 1e). The supernatant level of a Type-3-cytokine (IL-17) from mice sensitized with OVA in the presence and absence of chitin was not altered when compared with that from mice sensitized with chitin alone or saline (Fig. 1e). In addition, the levels of IL-5 and IL-13 in the BALFs were significantly increased in mice sensitized with OVA in the presence of chitin, but not in the other groups, after the last OVA challenge (Fig. 2a). On the other hand, the levels of IFN-γ and IL-17 in the BALFs were not increased in any of the groups (Fig. 2a). It is thought that Th2 cells and group 2 innate lymphoid cells (ILC2) may be potential sources of type 2 cytokines in the lungs. We next examined the detection of IL-13 producing cells in the lungs and BALFs in each group. We found that the proportion and number of IL-13^+^ Th2 cells were significantly increased in both the lungs and BALFs from mice sensitized with OVA in the presence of chitin compared with the other groups (Fig. 2b,c). Although IL-13^+^ ILC2 were significantly detected in the lungs, but not the BALFs, from mice sensitized with OVA in the presence of chitin compared with the other groups (Fig. 2b,c), the number of IL-13^+^ ILC2 was relatively smaller than that of IL-13^+^ Th2 cells (Fig. 2b,c). In addition, airway inflammation was not observed in T/B cell-deficient *Rag2*^−/−^ mice (Supplemental Fig. 1). These observations suggest that the major source of IL-13 in our model was Th2 cells rather than ILC2, although we could not rule out a possible contribution of ILC2 in the setting. Taken together, these observations strongly suggest that chitin, which was administered by the intranasal route, has the potential to exert an adjuvant effect on Type 2-cytokine-associated immune responses during OVA-induced airway inflammation.

**Figure 3:**
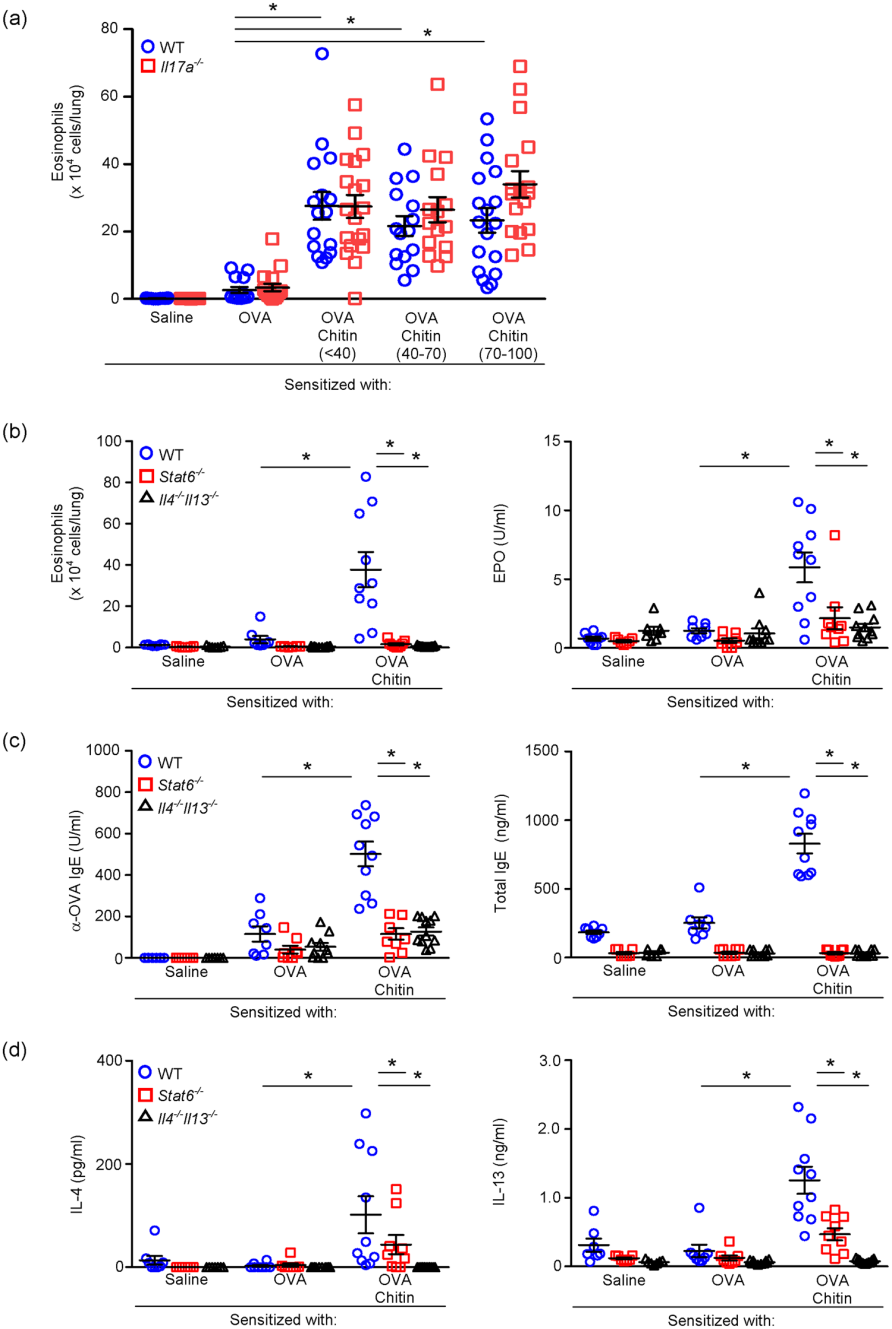
Chitin amplifies development of OVA-induced airway inflammation dependent on IL-4/13 and STAT6, but independently of IL-17. (**a**) The number of eosinophils in the BALFs from C57BL/6-wild-type and -*Il17a*^−/−^ mice intranasally sensitized with or without OVA ± different sizes of chitin particles and challenged with OVA, as shown in Fig. 1a. (**b**) The number of eosinophils and levels of EPO activities in the BALFs from BALB/c-wild-type, -*Stat6*^−/−^ and *Il4*^−/−^*Il13*^−/−^ mice sensitized with or without OVA ± chitin (<100 µm) and challenged with OVA, as shown in Fig. 1a. (**c**) Levels of OVA-specific and total IgE in sera from mice in B. (**d**) Levels of IL-4 and IL-13 in culture supernatants of spleen cells from mice in b after *in vitro* restimulation with OVA. Data are pooled from 2 independent experiments and shown as the mean ± SE. 1-way or 2-way ANOVA, *P < 0.05.

As in the case of alum, “intraperitoneal” administration of chitin particles facilitated OVA-induced airway inflammation^14^, but inhibited ragweed antigen-induced airway inflammation in mice^15^. The effects of chitin on T cell-mediated allergic inflammation may differ as a function of the size of its particles^9,10,14,15^. We thus investigated the effect of chitin particle size in our model, as shown in Fig. 1a. The number of eosinophils in the BALFs was similarly increased in wild-type mice sensitized with OVA in the presence of all sizes of chitin particles fractionated into <40 µm, 40–70 µm or 70–100 µm in diameter (Fig. 3a). These data show that any size of chitin particles less than 100 µm in diameter in our preparation has similar potency as an adjuvant of induction of allergic airway inflammation by sensitization with OVA via the intranasal route.

It was also reported that TLR2-dependent IL-17 production is important for development of OVA-induced airway inflammation in mice sensitized “intraperitoneally” with OVA in the presence of chitin particles (40- to 70-µm in diameter)^14^. Therefore, we examined the contribution of IL-17 to induction of OVA-induced airway inflammation in *Il17a*^−/−^ mice sensitized “intranasally” with OVA in the presence of various sizes of chitin particles. However, regardless of the chitin particle size, the number of eosinophils in BALFs from those mice was only slightly, without significance, increased compared with wild-type mice (Fig. 3a). These data suggest that IL-17 is not essential for induction of allergic airway inflammation in mice sensitized “intranasally” with OVA in the presence of various sizes of chitin particles. That finding suggests that different mechanisms underlie the adjuvant effects of chitin in the sensitization to OVA when administered intranasally and intraperitoneally. Therefore, we hereafter used <100-µm-diameter chitin particles to investigate the detailed mechanism of the adjuvant effect of chitin via the intranasal route.

It is well established that the development of airway inflammation induced by “intraperitoneal” sensitization with OVA emulsified with alum is dependent on the IL-4/IL-13-IL-4Rα-STAT-6 pathway^16,17^. On the other hand, it was also reported that airway eosinophilia was induced independently of the IL-4/IL-13-IL-4Rα-STAT-6 pathway in certain settings, i.e., sensitization with OVA in the presence of a high dose of dsRNA^18^ and inhalation of fungal antigens^19^. Therefore, it is important to clarify whether development of airway eosinophilia in mice sensitized with OVA in the presence of chitin requires IL-4/IL-13-IL-4Rα-STAT-6 signaling. Accordingly, we examined the role of that signaling in *Stat6*^−/−^ mice and *Il4*^−/−^ *Il13*^−/−^ mice. As expected, the eosinophil count and the EPO activity level in BALFs from wild-type mice sensitized with OVA in the presence of chitin were increased compared with wild-type mice sensitized with OVA in the absence of chitin or with saline alone (Fig. 3b). On the other hand, such increased responses were not observed in *Stat6*^−/−^ mice or *Il4*^−/−^ *Il13*^−/−^ mice (Fig. 3b). After the last challenge, the levels of total and OVA-specific serum IgE in wild-type mice, but not *Stat6*^−/−^ mice or *Il4*^−/−^ *Il13*^−/−^ mice, sensitized with OVA in the presence of chitin were markedly increased in comparison with in wild-type, *Stat6*^−/−^ mice and *Il4*^−/−^ *Il13*^−/−^ mice sensitized with OVA in the absence of chitin and/or with saline alone (Fig. 3c). In addition, the levels of IL-4 and IL-3 produced by OVA-stimulated spleen cells from *Stat6*^−/−^ and *Il4*^−/−^ *Il13*^−/−^ mice that had been sensitized with OVA in the presence of chitin were impaired and diminished, respectively, in comparison with wild-type mice (Fig. 3d). Taken together, it can be surmised that, in contrast to the special settings noted above^18,19^, IL-4/IL-13 and STAT-6 are required for induction of Type 2-cytokine-mediated immune responses in mice sensitized with OVA in the presence of chitin.

### IL-33, but not IL-25 or TSLP, is required for allergic airway inflammation in the presence of chitin

**Figure 4:**
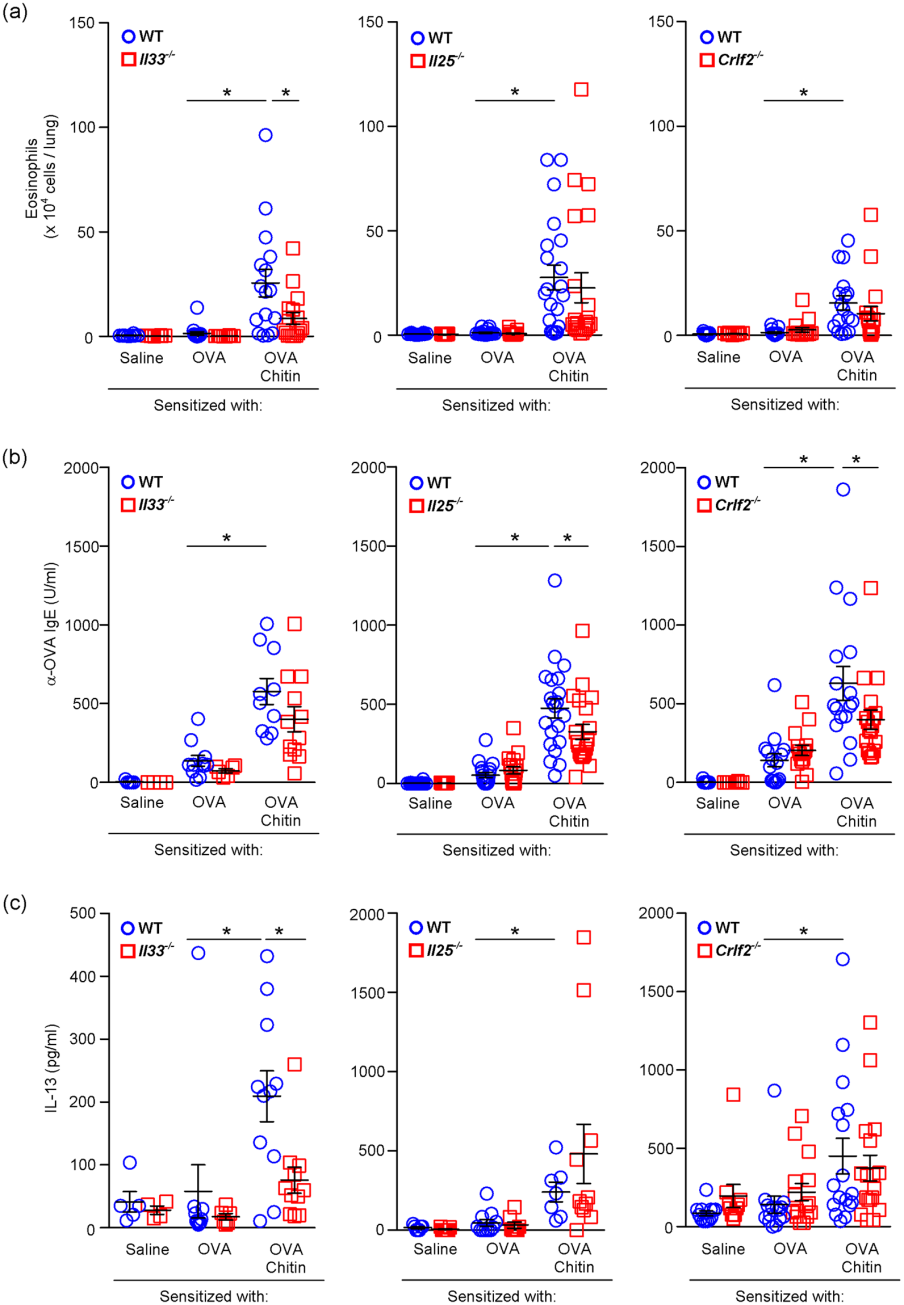
Chitin amplifies development of OVA-induced airway inflammation dependent on IL-33, but not IL-25 or TSLP. BALB/c-wild-type, -*Il33*^−/−^, -*Il25*^−/−^ and *Crlf2*^−/−^ mice were intranasally sensitized with or without OVA ± chitin and then challenged intranasally with OVA, as shown in Fig. 1a. (**a**) The number of eosinophils in the BALFs. (**b**) Levels of OVA-specific IgE in sera. (**c**) Levels of IL-13 in culture supernatants of spleen cells after *in vitro* restimulation with OVA. The data are pooled from at least 3 independent experiments and shown as the mean ± SE. 1-way or 2-way ANOVA, *P < 0.05.

IL-25, IL-33 and TSLP, which are considered to be produced by pulmonary epithelial cells, are well known to be involved in promoting Type-2 immune responses, including IL-4/IL-13-IL-4Rα-STAT-6-dependent allergic airway inflammation, through induction of Type-2 cytokine production by various types of cells and alteration of DC phenotypes^20,21^. In addition, these cytokines were increased in the lungs after inhalation of chitin alone^13^. In our model, after the last OVA challenge, the number of eosinophils in BALFs and the production of IL-13, but not IL-4 (data not shown), by OVA-stimulated spleen cells from *Il33*^−/−^, but not *Il25*^−/−^ or *Crlf2*^−/−^, mice sensitized with OVA in the presence of chitin were significantly reduced in comparison with wild-type mice (Fig. 4a,c). On the other hand, the levels of OVA-specific serum IgE were not altered in any of those strains of mice after the last OVA challenge (Fig. 4b). These observations indicate that IL-33, but not IL-25 and TSLP, is crucial for induction of OVA-induced airway eosinophilia in the presence of chitin.

### Chitin abolishes tolerogenic immune responses

**Figure 5:**
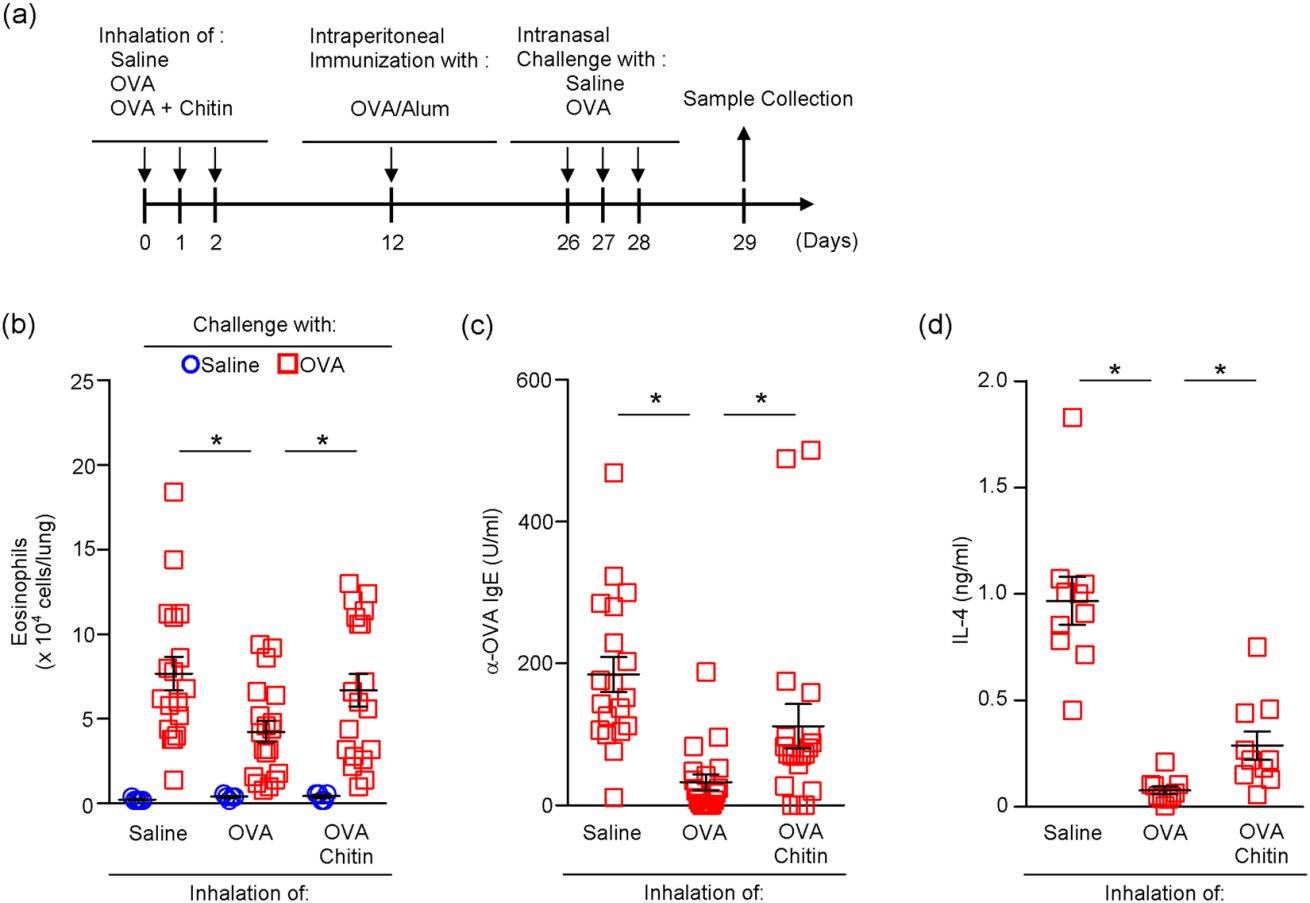
Chitin abolishes tolerogenic immune responses at airway. (**a**) Scheme of inhalation tolerance during OVA-induced airway inflammation. BALB/c-wild-type mice were intranasally exposed to OVA, OVA + chitin or saline prior to intraperitoneal sensitization with OVA/alum. After the sensitization, mice were challenged intranasally with OVA or saline. (**b**) The number of eosinophils in the BALFs. (**c**) Levels of OVA-specific IgE in sera. (**d**) Levels of IL-4 in culture supernatants of spleen cells after *in vitro* restimulation with OVA. Data are pooled from 4 independent experiments and shown as the mean ± SE. Mann–Whitney U test, *P < 0.05.

Inhalation of OVA prior to intraperitoneal sensitization with OVA emulsified with alum induces immune tolerance against OVA by generating tolerogenic pulmonary DCs, resulting in inhibition of development of OVA-induced airway inflammation by expansion of IL-10-producing Treg cells in mice^22^. On the other hand, the tolerance induction by inhalation of OVA is also known to be interfered with by persistent activation of pulmonary DCs by pathogens such as influenza virus^23,24^. Therefore, we investigated the effect of chitin on induction of tolerance by prior inhalation of OVA in mice (Fig. 5a). The number of eosinophils in BALFs, level of anti-OVA IgE in sera and production of IL-4 and IL-13 by OVA-stimulated spleen cells from wild-type mice that inhaled OVA prior to sensitization with OVA emulsified with alum was significantly reduced in comparison with wild-type mice that inhaled saline (Fig. 5b–d). On the other hand, interestingly, the tolerogenic responses induced by prior inhalation of OVA were partially but significantly restored in mice that inhaled OVA in the presence of chitin prior to sensitization with OVA emulsified with alum (Fig. 5b–d). These data suggest that chitin can promote immune responses by blocking induction of tolerogenic immune responses to harmless aero-antigens, leading to subsequent development of allergic airway inflammation.

### Chitin enhances OVA-induced airway eosinophilia by promoting IL-33-induced IL-1β production by DCs

**Figure 6:**
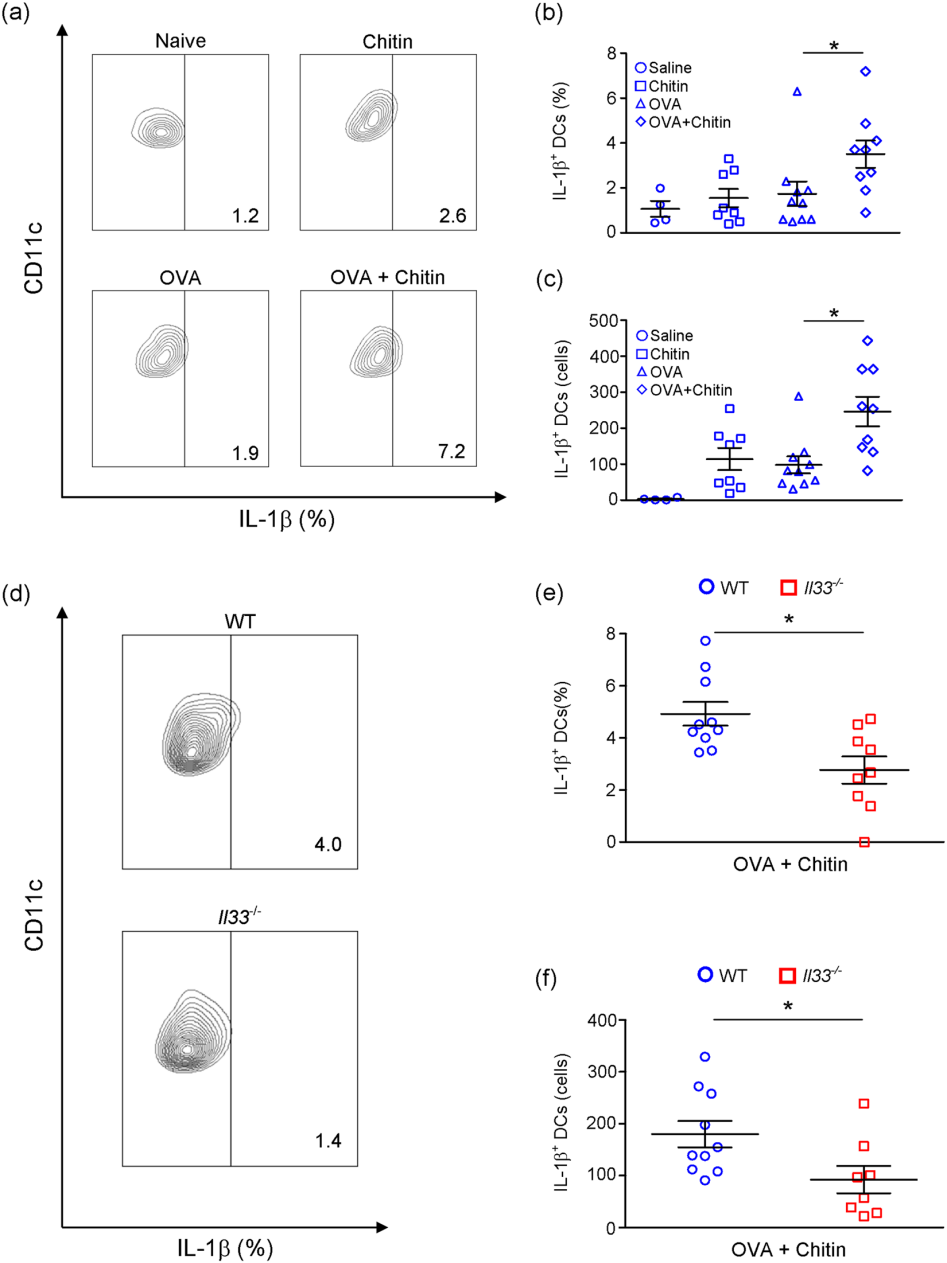
Chitin enhances activation of DCs dependent on IL-33. (**a**–**c**) BALB/c-wild-type mice, and (**d**–**f**) BALB/c-wild-type and -*Il33*^−/−^ mice were intranasally treated with chitin, OVA or OVA+ chitin on 3 consecutive days. Twenty-four hours after the last treatment, thoracic LNs were harvested. The proportion and number of CD45^+^ IL-1β-producing DCs in 7-AAD-negative I-A/I-E^hi^CD11c^hi^ cells in the LNs were determined by flow cytometry. Data are pooled from 4-independent (**a**–**c**) and 2-independent (**d**–**f**) experiments and shown as the mean ± SE. 1-way ANOVA or Mann–Whitney U test, *P < 0.05.

How can chitin break tolerance? It is known that IL-1 can break tolerance of T cells and B cells to certain antigens^25–27^. Therefore, we investigated the effect of chitin on IL-1β production by DCs after inhalation of chitin, OVA, or OVA + chitin. Pulmonary DCs migrated into draining LNs after inhalation of FITC-conjugated OVA^28,29^. We found that the proportion and number of IL-1β^+^ DCs among 7-AAD^−^ CD45^+^ MHCII^hi^ CD11c^+^ cells in draining LNs were comparable in each of naïve, OVA-treated and chitin-treated wild-type mice (Fig. 6a–c). On the other hand, that proportion and number were significantly increased in OVA + chitin-treated wild-type mice compared with the other groups (Fig. 6a–c), suggesting that chitin is a potent activator of DCs in the presence of OVA. IL-1β^+^ DCs could not be detected in *Il1a*^−/−^*Il1b*^−/−^ mice in the settings (Supplemental Fig. 2).

**Figure 7:**
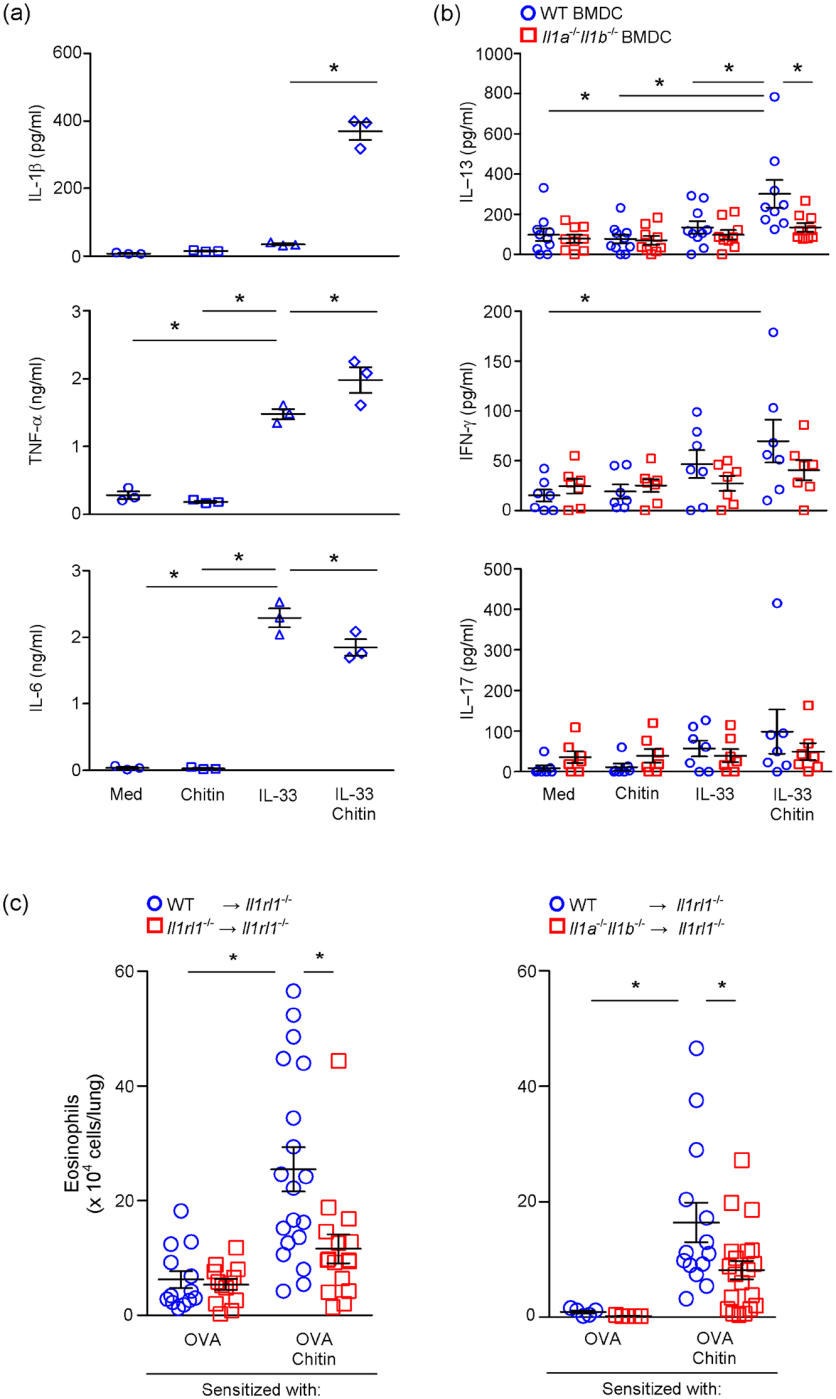
Chitin enhances OVA-induced airway eosinophilia by promoting Th2 cell activation dependent on augmentation of IL-33-induced IL-1β production by DCs. (**a**) BMDCs derived from C57BL/6-wild-type mice were cultured in the presence and absence of chitin, rIL-33 and rIL-33+ chitin for 24 h. The levels of IL-1β, IL-6 and TNF in the culture supernatants were assessed by ELISA. The data show the mean ± SE. Results are representative of similar results that were obtained in 3 independent experiments. 1-way ANOVA, *P < 0.05. (**b**) BMDCs from C57BL/6-wild-type and -*Il1a*^−/−^*Il1b*^−/−^ mice were cultured in the presence and absence of chitin, IL-33 and IL-33+ chitin for 24 h. After washing, the BMDCs were co-cultured with CD4^+^ T cells from OTII mice in the presence of OVA peptides for 4 days. The levels of IL-13, IL-17 and IFN-γ in the culture supernatants were assessed by ELISA. Data are pooled from 3 independent experiments and shown as the mean ± SE. 1-way or 2-way ANOVA, *P < 0.05. (**c**) Naive *II1rl1*^−/−^ mice were transplanted intranasally with DCs derived from C57BL/6-wild-type, - *II1rl1*^−/−^ or *Il1a*^−/−^*Il1b*^−/−^ mice. The mice were sensitized and challenged as in Fig. 1a. The number of eosinophils in the BALFs from these mice were determined. Data are pooled from 2–3 independent experiments and shown as the mean ± SE. 1-way ANOVA or Mann–Whitney U test, *P < 0.05.

As shown in Fig. 4, *Il33*^−/−^ mice showed attenuation of induction of OVA-induced airway eosinophilia in the presence of chitin, indicating that IL-33 is crucial in the setting. Interestingly, the proportion and number of IL-1β^+^ DCs among 7-AAD^−^ CD45^+^ MHCII^hi^ CD11c^+^ cells in draining LNs was significantly reduced in *Il33*^−/−^ mice compared with wild-type mice (Fig. 6d–f), suggesting that IL-33 induced by chitin may be involved in IL-1β production by DCs in the presence of OVA. To elucidate this, we cultured bone marrow-derived DCs (BMDCs) in the presence and absence of IL-33, with and without chitin. BMDCs produced IL-1β, IL-6 and TNF in response to IL-33, but not chitin (Fig. 7a). On the other hand, chitin enhanced IL-33-dependent IL-1β, but not IL-6 or TNF, production by BMDCs (Fig. 7a). These findings suggest that excessive IL-1β production by IL-33-stimulated DCs in the presence of chitin may be involved in the breaking of tolerance, resulting in excessive T-cell activation in response to allergens. Therefore, we next investigated the effect of IL-1β, produced by IL-33-stimulated BMDCs in the presence of chitin, on OVA-specific T-cell activation. BMDCs from wild-type mice were first incubated with or without IL-33 in the presence and absence of chitin, and then co-cultured with CD4^+^ T cells from OTII mice, which express OVA-specific TCR, in the presence of OVA peptides. As shown in Fig. 7b, the level of IL-13, but not IL-17 or IFN-γ, in the culture supernatants of the CD4^+^ OTII cells co-cultured with IL-33- and chitin-stimulated wild-type BMDCs were significantly increased compared with unstimulated, IL-33-stimulated and chitin-stimulated wild-type BMDCs. However, IL-13 production was not increased in the supernatants of the CD4^+^ OTII cells co-cultured with IL-33- or the chitin-stimulated *Il1a*^−/−^*Il1b*^−/−^ BMDCs (Fig. 7b). These observations suggest that chitin can promote IL-33-induced IL-1β production by DCs, followed by enhancement of antigen-specific Th2-cell activation, *in vitro*.

In order to elucidate whether IL-1β production by DCs in response to IL-33 induced by chitin is important for induction of allergic airway eosinophilia *in vivo*, we established OVA-induced airway eosinophilia in the presence of chitin in *Il1rl1*^−/−^ mice transferred with wild-type or *Il1rl1*^−/−^ DCs (wild-type DCs → *Il1rl1*^−/−^ mice; or *Il1rl1*^−/−^ DCs → *Il1rl1*^−/−^ mice), or with wild-type or *Il1*^−/−^ DCs (wild-type DCs → *Il1rl1*^−/−^ mice; or *Il1*^−/−^ DCs → *Il1rl1*^−/−^ mice), as shown in Fig. 1a. The number of eosinophils in BALFs was significantly increased in wild-type DCs → *Il1rl1*^−/−^ mice sensitized with OVA in the presence of chitin in comparison with wild-type DCs → *Il1rl1*^−/−^ mice sensitized with OVA alone at 24 h after the last OVA inhalation (Fig. 7c). On the other hand, the number of eosinophils in BALFs from *Il1rl1*^−/−^ DCs → *Il1rl1*^−/−^ mice was significantly reduced in comparison with wild-type DCs → *Il1rl1*^−/−^ mice in the setting (Fig. 7c). These observations indicate that activation of IL-1RL1/ST2-expressing DCs in response to IL-33 induced in the lungs after respiratory exposure to chitin is crucial for induction of OVA-induced airway eosinophilia. The number of eosinophils was also reduced in BALFs from *Il1a*^−/−^*Il1b*^−/−^ DCs → *Il1rl1*^−/−^ mice compared with wild-type DCs → *Il1rl1*^−/−^ mice (Fig. 7c). These observations indicate that chitin-induced IL-33 stimulates DCs to produce IL-1β, which is involved in induction of OVA-induced airway eosinophilia *in vivo*.

## Discussion

Chitin, β-(1–4)-poly-N-acetyl D-glucosamine, is known to be a common component of the exoskeleton of arthropods, such as house dust mites, crabs, shrimp and insects, and the microfilarial sheath of parasitic nematodes^1–5^. It was reported that inhalation of chitin alone resulted in induction of airway eosinophilia in mice^11,13^, suggesting that chitin may be somehow involved in the pathogenesis of HDM-mediated asthma. Induction of type 2-cytokine-mediated airway inflammation by ragweed antigen was inhibited by intraoral administration of chitin particles (1- to 10-µm in diameter) in the antigen sensitization phase by enhancing IFN-γ production^15^. Development of type 2-cytokine-mediated eosinophilia and IL-17-mediated neutrophilia during OVA-induced airway inflammation was promoted by intraperitoneal administration of chitin particles (40- to 70-µm in diameter) in the antigen sensitization phase by promoting IL-17 production by macrophages via TLR2 and/or Dectin-1^9,10,14^. Those findings suggest that the effects of chitin on T cell-mediated allergic inflammation differ as a function of the size of chitin particles.

IL-33, IL-25 and TSLP, which are preferentially produced by lung epithelial cells, are considered to be involved in development of type 2 cytokine-associated allergic airway inflammation^20,30–32^. They have been reported to be crucial for induction of OVA-induced airway inflammation in mice sensitized “intraperitoneally” with OVA in the presence of alum^29,33,34^. On the other hand, only IL-33, but not IL-25 or TSLP, was important for induction of HDM-induced airway inflammation in mice sensitized “intranasally” with HDM^35,36^. Similar to the latter observation, we demonstrated that IL-33, but not IL-25 or TSLP, is responsible for induction of OVA-induced airway inflammation in mice sensitized “intranasally” with OVA in the presence of chitin.

**Figure 8:**
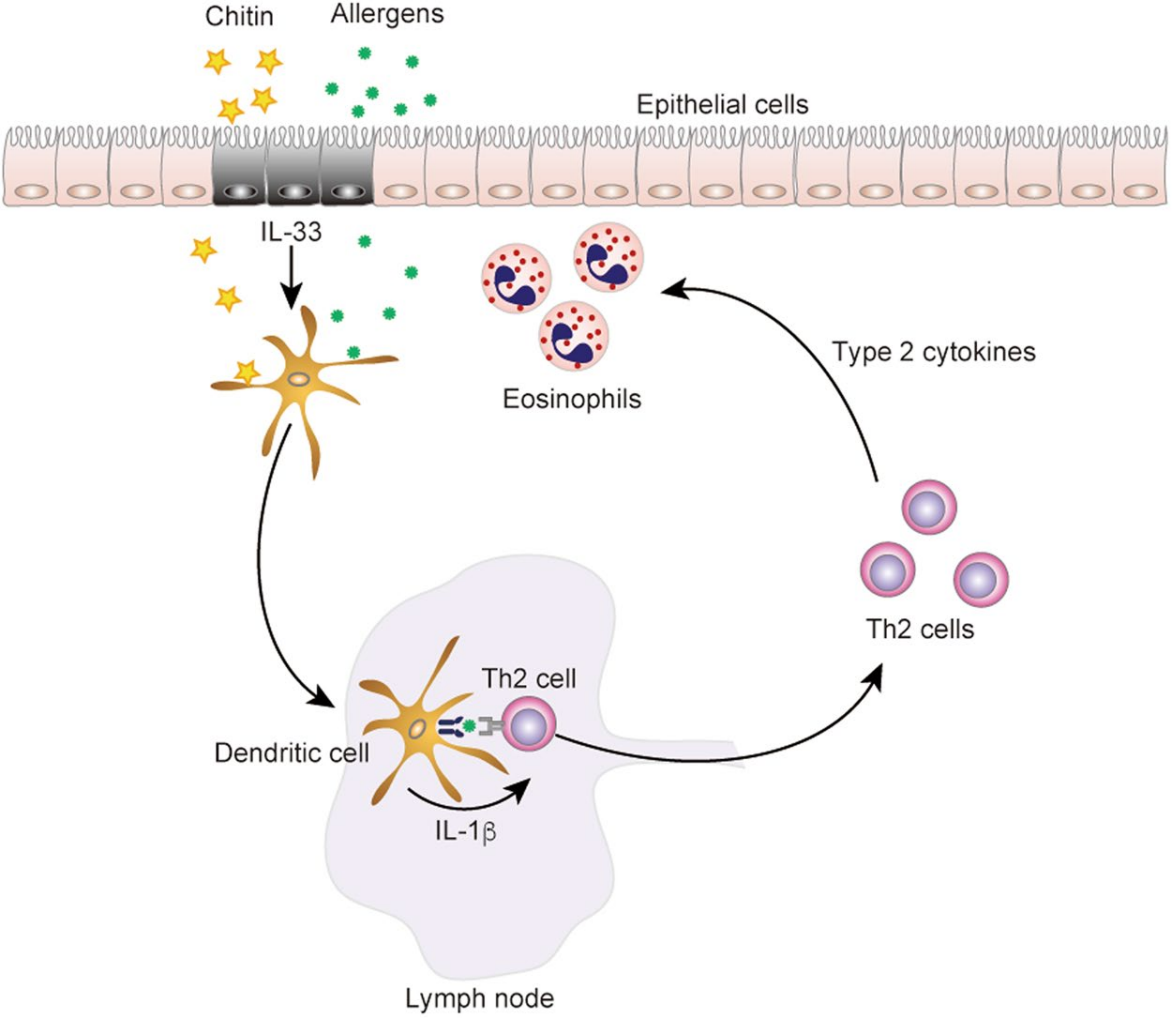
Schematic representation of the effect of chitin on antigen-specific Th2 cell-mediated murine asthma. Intranasal administration of chitin induced IL-33 in the lungs, and IL-1β produced by DCs in response to that IL-33 promotes Th2 cell activation, resulting in aggravation of OVA-induced type 2-cytokine-mediated allergic airway eosinophilia that is dependent on an IL-4/IL-13-STAT6 pathway.

In addition, in the present study, we found that the administration route of chitin affects the development of OVA-induced airway inflammation. We demonstrated that chitin administered intranasally has the potential to exert an adjuvant effect on Type 2-cytokine-associated immune responses during OVA-induced airway inflammation. Especially, the mechanisms for the effect of chitin via the intranasal route differed from those via the intraperitoneal route, as reported previously^14^. That is, the mechanisms for the effect of chitin via the intranasal route are mediated in an IL-4/IL-13-STAT6-dependent, but IL-17A-independent, manner. On the other hand, it was reported that the mechanisms via the intraperitoneal route are mediated in an IL-17A-dependent manner^14^. In addition, inhalation of chitin in mice resulted in increased IL-33 expression in the lungs^13^. In association with this, we found that 1) IL-33 induced IL-1β production by DCs, 2) chitin promoted IL-33-dependent IL-1β production by DCs, and 3) DC-derived IL-1β enhanced antigen-specific Th2 cell activation (Fig. 8), contributing to induction of Th2 cell-mediated IL-4/IL-13-STAT6-dependent allergic airway inflammation.

IL-33 was reported to induce cytokine production (i.e., IL-1β, IL-6 and TNF) and enhance co-stimulatory molecule expression (i.e., CD40, CD80 and OX40L) in DCs, followed by enhancement of Th2 cell differentiation via that activation of DCs^37^. In addition, adoptive transfer of OVA-pulsed, IL-33-stimulated DCs resulted in aggravated OVA-induced airway inflammation compared with OVA-pulsed, unstimulated DCs in mice^37^. We found that chitin alone did not induce cytokine production (i.e., IL-1β, IL-6 and TNF), but it enhanced IL-33-induced IL-1β production, by DCs (Fig. 6a). On the other hand, chitin did not enhance CD40 or OX40L expression on IL-33-stimulated DCs (data not shown). It is known that IL-1 can break the tolerance of T cells and B cells to certain antigens^25–27^, suggesting that excessive IL-1β production by DCs in response to IL-33 in the presence of chitin may be involved in breaking the tolerance of T cells to allergens, such as OVA used in this study. It is well known that respiratory exposure to allergens leads to tolerance of T cells to those allergens, contributing to protection against induction of allergic asthma through induction of tolerogenic pulmonary DCs in non-atopic individuals^22^. On the other hand, it was reported that such tolerance to allergens was canceled by concurrent viral infection, such as influenza A, resulting in provocation of allergic airway inflammation^23,24^. Likewise, we found that inhalation tolerance to OVA in a mouse model^22^ was abolished by concurrent inhalation of chitin. We also demonstrated that IL-1β derived from DCs in response to IL-33 and chitin is important for Th2 cell activation in the sensitization phase, which is involved in aggravation of allergic airway inflammation.

In conclusion, we demonstrated that intranasal administration of chitin induced IL-33 in the lungs, and IL-1β production by DCs in response to IL-33 is important for Th2 cell polarization, resulting in aggravation of OVA-induced Th2 cell-mediated allergic airway inflammation that is dependent on an IL-4/IL-13-STAT6 pathway. These observations suggest that intranasal exposure to chitin can potentially act as a type 2-cytokine-associated natural adjuvant, contributing to the pathogenesis of allergic disorders induced by a variety of allergens, including HDM.

## Methods

### Mice

BALB/c- and C57BL/6-wild-type mice were obtained from Japan SLC. *Il4*^−/−^ *Il13*^−/−^ mice^38^, *Stat6*^−/−^ mice^39^, *Il33*^−/−^ mice^34^, *Il25*^−/−^ mice^40^ and *Crlf2*^−/−^ mice^41^ on the BALB/c background, and *Il17a*^−/−^ mice^42^, *Il1a*^−/−^*Il1b*^−/−^ mice^43^, *Il1rl1*^−/−^ mice^44^, MHCII-restricted OVA-TCR Tg OTII mice^45^ and *Il13*-reporter mice (*Il13*^tomato/+^)^46^ on the C57BL/6 background were used. *Rag2*^−/−^ mice on the C57BL/6 background were kindly provided by Dr. Hiromitsu Nakauchi (The University of Tokyo, Japan). Six- to 12-week-old mice (both females and males) were used in the experiments. All mice were housed under specific pathogen-free conditions in an environmentally-controlled animal room at the National Research Institute for Child Health and Development and the Faculty of Health Sciences, Kyorin University. All animal experiments were approved and performed in accordance with the animal care and use committees of Kyorin University (I16-01-02), the National Research Institute for Child Health and Development (A2012-004-C05) and the Institute of Medical Science, The University of Tokyo (A11-28).

### Preparation of chitin suspension

Chitin (Dextra Laboratories) was treated and reduced in size using an established procedure^47^. In brief, chitin was partially hydrolyzed with concentrated hydrochloric acid for 30 min at 30 °C, followed by neutralization with 1 M Tris-HCl buffer (pH 8.0) and centrifugation at 3,000 rpm for 5 min at room temperature. After washing with saline, the chitin was routinely passed through 100-µm nylon mesh filter, or fractionated by size-exclusion nylon mesh filters into 70–100 µm, 40–70 µm and <40 µm diameter particles. The chitin was resuspended in saline at the appropriate concentration, sterilized by autoclaving and stored at 4 °C.

### Animal model of allergic asthma

As shown in Fig. 1a, mice were intranasally treated with 10 µg OVA (grade V; Sigma) in saline in the presence and absence of 10 µg chitin in 20 µl of saline or saline alone on each of seven alternate days (days 0, 2, 4, 6, 8, 10 and 12). On days 28, 31 and 34, the mice were intranasally challenged with 100 µg OVA in 20 µl of saline. One day after the last challenge, sera, lungs, bronchoalveolar lavage fluids (BALFs) and spleens were harvested.

### Bronchoalveolar lavage (BAL) cell analysis

BALFs were collected as described elsewhere^48^. The total cell count and leukocyte profile were determined with a hemocytometer (XT-1800i; Sysmex), as described previously^34^.

### Histology

Lungs were harvested, fixed in 10% neutral buffered formalin and embedded in paraffin. Three-µm-thick lung sections were stained with hematoxylin and eosin (HE).

### ELISA for immunoglobulins

OVA-specific IgE, IgG_1_ and IgG_2a_ levels in sera were determined by ELISA, as described elsewhere^34^. The data were normalized to the value for arbitrary serum obtained from OVA-immunized mice as a standard calibrator. Mouse IgE ELISA Quantitation Kit (Bethyl Laboratories) was used to measure the total IgE in sera.

### Spleen cell culture

After elimination of RBCs with an RBC lysing buffer (Sigma), spleen cells were suspended in RPMI1640 medium supplemented with 10% FBS, 100 U/ml penicillin and 100 µg/ml streptomycin. Spleen cells (4 × 10^6^ cells/ml) were cultured in the presence of 100 µg/ml OVA in 24-well flat-bottom plates for 4 d.

### ELISA for cytokines

The levels of IFN-γ, IL-4, IL-13, IL-17A, IL-1β, TNF and IL-6 in the cell culture supernatants and BALFs were determined by ELISA using mouse IFN-γ, IL-4, IL-5, IL-13, IL-17A, IL-1β, TNF-α and IL-6 ELISA Ready-SET-Go!^®^ (eBioscience). All procedures were performed according to the manufacturer’s instructions.

### Eosinophil peroxidase assay

The level of eosinophil peroxidase (EPO) activity in the BALFs was examined as described previously^34^.

### Inhalation tolerance model

An animal model of tolerogenic immune responses induced by respiratory exposure to antigen was previously reported^22^. In brief, mice were exposed intranasally to 100 μg of OVA in 20 μl of saline in the presence and absence of 100 μg of chitin (<100 µm-diameter), or saline alone, on 3 consecutive days as shown in Fig. 4a. Ten days after the last inhalation, the mice were immunized intraperitoneally with 10 μg of OVA emulsified in 2 mg of alum hydroxide (alum). Two weeks later, the mice were challenged with 50 μg of OVA in 20 μl of saline, or saline alone, intranasally on 3 consecutive days. One day after the last challenge, sera, BALFs and spleens were harvested.

### FACS analysis

For detection of IL-13^+^ cells, *Il13*-reporter (*Il13*^tomato/+^) mice were intranasally sensitized with saline, chitin, OVA or OVA + chitin, and challenged with OVA, as shown in Fig. 1a. After the last OVA challenge, the lungs and BALFs were collected. The lungs were minced and digested in PBS containing 1% BSA, 200 U/ml collagenase type 5 (Worthington Biochemical Corporation), 1500 U/ml hyaluronidase (Worthington Biochemical Corporation) and 100 U/ml DNase I (Worthington Biochemical Corporation) for 30 min at 37 °C. After the digested lungs were homogenized with gentleMACS^TM^ Dissociator (Miltenyi Biotec), single lung cells were collected by passing through a 100-µm nylon mesh. The single lung cells and BAL cells were incubated with anti-mouse CD16/CD32 mAb (93; BioLegend) in FACS buffer (HBSS containing 2% FCS) for FcR blocking for 20 min on ice. The cells were then incubated (30 min on ice) with a mixture containing FITC-conjugated anti-mouse ST2 mAb (DJ8; MD Biosciences), PE-Cy7-conjugated anti-mouse CD127 mAb (A7R34; BioLegend), BD Horizon^TM^ BV421-conjugated anti-mouse KLRG1(Killer cell Lectin-like Receptor G1) mAb (2F1/KLRG1; BioLegend), BD Horizon^TM^ BV510-conjugated anti-mouse CD45 mAb (30-F11; BioLegend), APC-conjugated anti-mouse Sca-1 mAb (D7; BioLegend), PerCP-Cy5.5-conjugated anti-mouse CD3ε mAb (145–2C11; BioLegend), Alexa Fluor^®^ 700-conjugated anti-mouse CD4 mAb (GK1.5; BioLegend), biotin-conjugated anti-mouse lineage marker mAbs (FcεRIα [MAR-1; BioLegend], CD11c [N418; BioLegend], CD19 [6D5; BioLegend], NK1.1 [PK136; BioLegend], F4/80 [BM8; BioLegend], Ter119 [TER-119; BioLegend], Gr-1 [RB6-8C5; BioLegend], CD5 [53-7.3; BioLegend]], and eBioscience^TM^ Fixable Viability Dye eFluor^TM^ 780 (Thermo Fisher Scientific). After washing, the proportion of IL-13-tomato^+^ cells among Viability Dye-negative CD45^+^ CD3ε^+^ CD4^+^ cells (as activated Th2 cells) and CD45^+^ CD3ε^−^ CD4^−^ lineage^−^ Sca-1^+^ KLRG1^+^ CD127^+^ ST2^+^ cells (as activated ILC2) were analyzed on a FACS Aria II Cell Sorter (BD Biosciences) with BD FACSDiva software and FlowJo software (Tree Star).

For detection of IL-1β^+^ cells, mice were exposed intranasally to 100 μg of OVA in 20 μl of saline in the presence and absence of 100 μg of chitin (<100 µm diameter), or saline alone, on 3 consecutive days. Thoracic lymph nodes (LNs) were collected 24 h after the last inhalation, and single-cell suspensions were prepared as described elsewhere^28,49^. LN cells were cultured in RPMI1640 medium supplemented with 10% FBS, 100 U/ml penicillin and 100 μg/ml streptomycin at 37 °C for 3 h. The cells were incubated with anti-mouse CD16/CD32 mAb in FACS buffer for FcR blocking for 20 min on ice and then incubated with a mixture of APC-Cy7-conjugated anti-mouse CD45 mAb (30-F11; BioLegend), FITC-conjugated anti-mouse I-A/I-E mAb (M5/114.15.2; BioLegend) and PE-Cy7-conjugated anti-mouse CD11c mAb (N418; BioLegend) for 30 min on ice. After washing, the cells were fixed with Phosflow^TM^ Fix Buffer I (BD Bioscience), permeabilized with 0.1% saponin in FACS buffer and incubated with PE-conjugated anti-mouse IL-1β (Pro) mAb (NJTEN3; BioLegend) at 4 °C for 30 min. The proportion of IL-1β^+^ cells among 7-aminoactinomycin D-negative CD45^+^I-A/I-E^hi^CD11c^hi^ cells in LN cells was analyzed on a FACS Aria II Cell Sorter with BD FACSDiva software and FlowJo software.

### BMDCs

BM cells were isolated by flushing femurs and tibiae with HBSS (Invitrogen). The BM cells were then passed through a 70-μm nylon mesh filter, centrifuged and resuspended in RBC lysing buffer at room temperature for 5 min to lyse RBCs. The cells (2 × 10^5^ cells/ml in ϕ 9 cm serological dishes) were cultured in the presence of 20 ng/ml of recombinant mouse GM-CSF (rmGM-CSF; Peprotech) in RPMI 1640 medium supplemented with 10% FBS, 100 U/ml of penicillin and 100 μg/ml of streptomycin at 37 °C. On days 5 and 9, the medium containing rmGM-CSF was refreshed. On day 12, non-adherent cells were harvested, and the CD11c^+^ cells contained therein were enriched (>90%) as BMDCs using CD11c MicroBeads (Miltenyi Biotec). BMDCs (1 × 10^6^ cells/well in flat-bottom 48-well plates) were incubated with and without 500 ng/ml of recombinant human IL-33 (rhIL-33; Peprotech), 1 mg/ml of chitin (<100 µm diameter), or 500 ng/ml of rhIL-33+ 1 mg/ml of chitin at 37 °C for 24 h.

### DC and T cell co-culture

BMDCs (2 × 10^6^ cells/ml) derived from WT and *Il1a*^−/−^ *Il1b*^−/−^ mice were incubated with and without 500 ng/ml of rhIL-33, 1 mg/ml of chitin, or 500 ng/ml of rhIL-33+ 1 mg/ml of chitin at 37 °C for 24 h. After removal of chitin by gradient centrifugation using lymphocyte separation medium (Wako Pure Chemical Industries), the BMDCs were purified using CD11c MicroBeads, as described above. CD4^+^ T cells from spleens of OTII mice (>90%) were purified magnetically by negative selection using a CD4^+^ T cell isolation kit II (Miltenyi Biotec). The BMDCs (2 × 10^4^ cells/well) were then co-cultured with CD4^+^ OTII T cells (2 × 10^5^ cells/well) in the presence of 1 nM OVA peptides in 96-well round-bottom plates at 37 °C for 4 days.

### BMDC transfer

BMDC transfer was performed as described elsewhere^37^, with minor modifications. In brief, naive *Il1rl1*^−/−^ mice were intranasally administered 20 µl of BMDCs (1 × 10^5^ cells in 20 µl saline) derived from WT mice, *Il1rl1*^−/−^ mice or *Il1a*^−/−^ *Il1b*^−/−^ mice. One day after the BMDC transfer, these mice were treated with antigens, as shown in Fig. 1a.

### Statistical analysis

Data show the mean ± standard error (SE). One-way or two-way ANOVA, or the Mann–Whitney U test was used for statistical evaluation using Graphpad Prism software (Graphpad Prism). P values of less than 0.05 were considered statistically significant.

### Data availability

All data generated or analyzed during this study are included in this published article and its Supplementary Information files.

## Electronic supplementary material

Supplemental Information
