# Supplementary material for: Chitin promotes antigen-specific Th2 cell-mediated murine asthma through induction of IL-33-mediated IL-1β production by DCs
Source: Sci Rep. 2018 Aug 6;8:11721. doi: 10.1038/s41598-018-30259-2 (PMC6079063; doi:10.1038/s41598-018-30259-2)
Supplement: Supplementary file 1 — Supplemental Information [file 41598_2018_30259_MOESM1_ESM.pdf]

# Chitin promotes antigen-specific Th2 cell-mediated murine asthma through induction of IL-33-mediated IL-1β production by DCs

Ken Arae[1,2], Hideaki Morita[2], Hirotoshi Unno[2], Kenichiro Motomura[2], Sumika Toyama[2], Naoko Okada[2], Tatsukuni Ohno[3], Masato Tamari[2], Keisuke Orimo[2], Yuko Mishima[1], Hajime Suto[4], Ko Okumura[4], Katsuko Sudo[5], Hiroshi Miyazawa[6], Haruhiko Taguchi[1], Hirohisa Saito[2], Kenji Matsumoto[2], and Susumu Nakae[7,8,*]

[1]Department of Immunology, [6]Department of Medical technology, Faculty of Health Sciences, Kyorin University, Tokyo, 181-8612, Japan; [2]Department of Allergy and Clinical Immunology, National Research Institute for Child Health and Development, Tokyo, 157-8535, Japan; [3]Department of Molecular Immunology, Graduate School of Medical and Dental Sciences, Tokyo Medical and Dental University, Tokyo, 113-8510, Japan; [4]Atopy Research Center, Juntendo University, Tokyo, 113-0033 Japan; [5]Animal Research Center, Tokyo Medical University, Tokyo, 160-8402, Japan; [7]Laboratory of Systems Biology, Center for Experimental Medicine and Systems Biology, The Institute of Medical Science, The University of Tokyo, Tokyo, 108-8639, Japan; [8]Precursory Research for Embryonic Science and Technology (PREST), Japan Science and Technology Agency, Saitama, Japan.

## Supplementary Figures

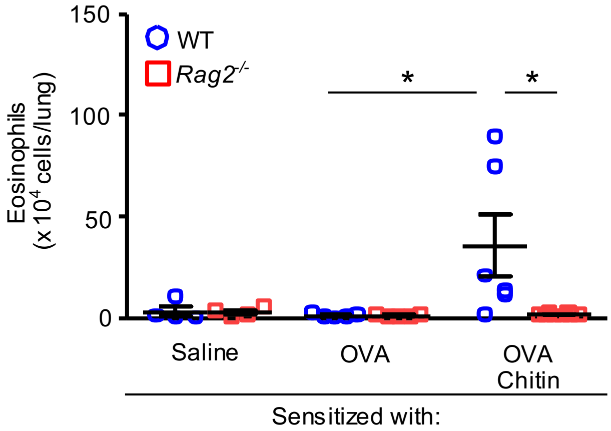


**Supplemental Figure 1.** OVA-specific T cells are essential for development of OVA-induced airway inflammation in the presence of chitin. The number of eosinophils in the BALFs from C57BL/6-wild-type and -*Rag2*$^{-/-}$ mice intranasally sensitized with or without OVA ± chitin and challenged with OVA, as shown in Fig. 1a. Data are shown as the mean ± SE. 1-way or 2-way ANOVA, **P* < 0.05.

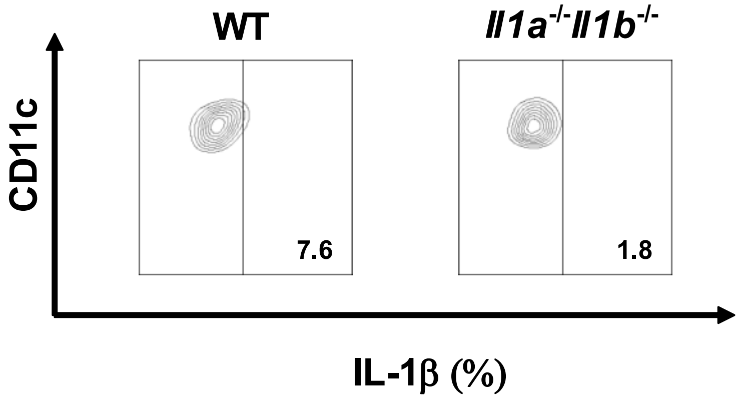


**Supplemental Figure 2.** Chitin induces IL-1β by DCs. C57BL/6-wild-type (n=3) and –*Il1a*$^{-/-}$*Il1b*$^{-/-}$ (n=3) mice were intranasally treated with OVA+Chitin on 3 consecutive days. Twenty-four hours after the last treatment, thoracic LNs were harvested. The proportion of CD45$^{+}$ IL-1β-producing DCs in 7-AAD-negative I-A/I-E$^{hi}$CD11c$^{hi}$ cells in the LNs was determined by flow cytometry. Data are shown as a representative data from 3-independent experiments.
